# New aspects in digital breast assessment: further refinement of a method for automated digital anthropometry

**DOI:** 10.1007/s00404-020-05862-2

**Published:** 2020-11-12

**Authors:** Robin Hartmann, Maximilian Weiherer, Daniel Schiltz, Magnus Baringer, Vivien Noisser, Vanessa Hösl, Andreas Eigenberger, Stephan Seitz, Christoph Palm, Lukas Prantl, Vanessa Brébant

**Affiliations:** 1 University Center of Plastic, Aesthetic, Hand and Reconstructive Surgery, University Hospital Regensburg, Franz-Josef-Strauß-Allee 11, 93053 Regensburg, Germany; 2 Regensburg Medical Image Computing (ReMIC), Ostbayerische Technische Hochschule Regensburg (OTH Regensburg), Regensburg, Germany; 3 Department of Obstetrics and Gynecology, Caritas Hospital St. Josef, University of Regensburg, Regensburg, Germany; 4 Regensburg Center of Biomedical Engineering (RCBE), OTH Regensburg and Regensburg University, Regensburg, Germany; 5 Faculty of Mechanical Engineering, Ostbayerische Technische Hochschule Regensburg (OTH Regensburg), Regensburg, Germany

**Keywords:** Three-dimensional imaging, Digital anthropometry, Breast symmetry, Breast surgery

## Abstract

**Purpose:**

In this trial, we used a previously developed prototype software to assess aesthetic results after reconstructive surgery for congenital breast asymmetry using automated anthropometry. To prove the consensus between the manual and automatic digital measurements, we evaluated the software by comparing the manual and automatic measurements of 46 breasts.

**Methods:**

Twenty-three patients who underwent reconstructive surgery for congenital breast asymmetry at our institution were examined and underwent 3D surface imaging. Per patient, 14 manual and 14 computer-based anthropometric measurements were obtained according to a standardized protocol. Manual and automatic measurements, as well as the previously proposed Symmetry Index (SI), were compared.

**Results:**

The Wilcoxon signed-rank test revealed no significant differences in six of the seven measurements between the automatic and manual assessments. The SI showed robust agreement between the automatic and manual methods.

**Conclusion:**

The present trial validates our method for digital anthropometry. Despite the discrepancy in one measurement, all remaining measurements, including the SI, showed high agreement between the manual and automatic methods. The proposed data bring us one step closer to the long-term goal of establishing robust instruments to evaluate the results of breast surgery.

**Level of evidence**: IV.

## Introduction

Objective evaluation of the aesthetic results for women undergoing breast surgery still presents a major challenge. Even today, manual anthropometric measurement is considered the most state-of-the-art technique for aesthetic breast assessment by clinicians [1–3].

In the last 2 decades, three-dimensional (3D) breast assessment has witnessed rapid progress [4–8]. Numerous protocols have been outlined using 3D surface imaging for evaluation of the results of breast surgery [9–12]. Nevertheless, the field of 3D surface imaging still lacks a sophisticated automated method for assessing breast aesthetics. Therefore, we developed software to assess these aesthetic results after breast surgery using automated anthropometry.

To further refine and validate this method we developed previously, we tested the prototype software by comparing anthropometric measurements obtained manually and digitally in patients who underwent reconstructive surgery for congenital breast asymmetry.

## Materials and methods

Before participant recruitment, the local ethics committee approved the study. The proposed method was tested in patients who underwent reconstructive surgery for congenital breast asymmetry at our institution from June 2008 to January 2019. The participants were invited to undergo a routine examination. Twenty-three patients who had undergone different reconstruction procedures agreed to be examined. Every patient underwent 3D surface imaging. For every patient, 14 anthropometric measurements were obtained manually using a tape measure, and 14 measurements were obtained automatically using the 3D data by our software, which has been described elsewhere [13].

### 3D surface imaging

The Vectra H2™ system (Canfield Scientific, USA) was used to reproduce the patient’s surface information. The portable photogrammetry scanning system has shown reliable results in assessing the human body [14]. It comes with Vectra Breast-Sculptor™ (Canfield Scientific, USA) and Vectra VAM™ (Canfield Scientific, USA) software for point cloud processing and mesh generation.

Photogrammetry is a well-validated method for assessing the human body [15–17]. The technique has shown robust results in capturing the human breast and is considered a valuable tool for surgical planning and evaluation in breast surgery.

The H2 system captures surface information as well as texture information. The result is a precise model of the human body. For breast assessment, a frontal 180° 3D model was generated using Vectra-Breast Sculptor™ (Canfield Scientific, USA), which is cut bilaterally at the level of the mid-upper brachium, the inguinal region and approximately at the thyroid cartilage. The model was then cut manually at the abdomen above the umbilicus using Vectra VAM™ software (Canfield Scientific, USA).

### Landmarks

**Fig. 1:**
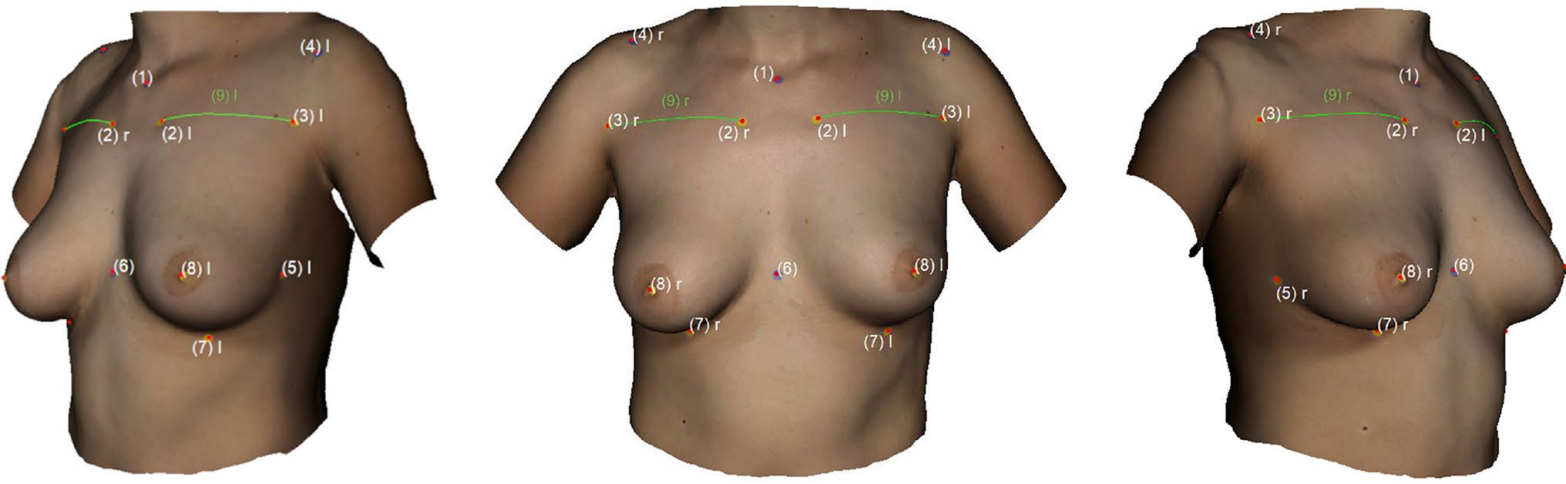
Landmarks (1)–(8); Appearance of a 22-year-old patient 2 years after lipofilling (L) in three stages; total transplanted volume, 540 cc (L); (1) Sternal Notch (SN), (2) Medial Upper Breast Pole (MUBP), (3) Lateral Upper Breast Pole (LUBP), (4) Coracoid process (CP), (5) Lateral Breast Pole (LaBP), (6) Xiphoid (Xi), (7) Lower Breast Pole (LBP), (8) Nipple (N), (9) Upper Breast Pole (UBP)

The following landmarks were marked: (1) Sternal Notch (SN), (2) Medial Upper Breast Pole (MUBP), (3) Lateral Upper Breast Pole (LUBP), (4) Coracoid process (CP), (5) Lateral Breast Pole (LaBP), (6) Xiphoid (Xi), (7) Lower Breast Pole (LBP), (8) Nipple (N), (9) Upper Breast Pole (UBP). Figure 1 provides an overview of all the landmarks.

The previous protocol [13] required 360° body scans to determine the vertebra prominens as a landmark. In the improved version of our software, the sagittal plane is determined using just 180° scans. This could be achieved by a manner similar to that described by Eder et al. [11], where the sagittal plane is defined through landmarks (1) and (6) and the midpoint between both points.

### Anthropometric measurements

**Fig. 2:**
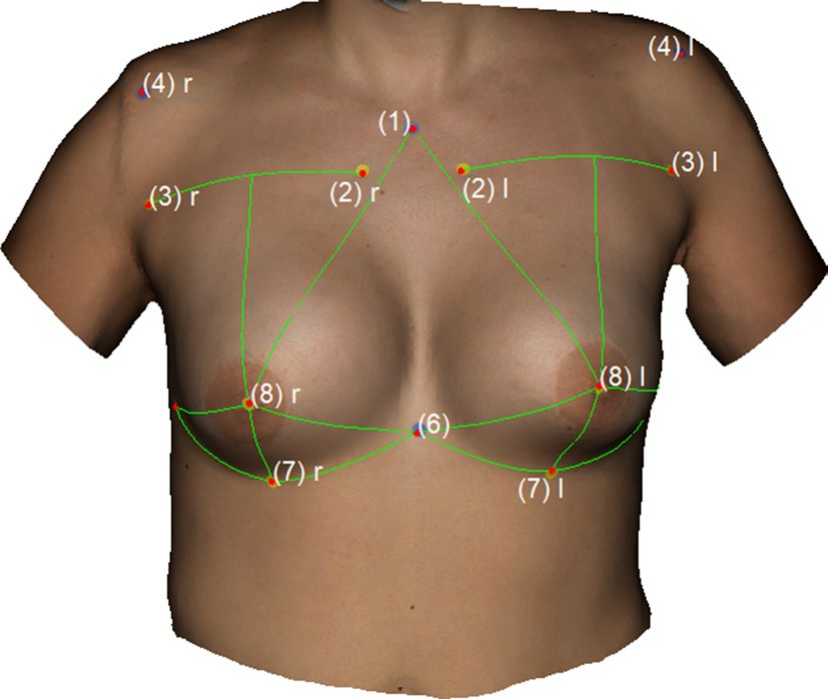
Automatic measurements. Appearance of a 32-year-old patient 12 years after bilateral permanent silicone gel breast implant installation; 225 cc (R)/200 cc (L); mastopexy R + L; frontal view

**Fig. 3:**
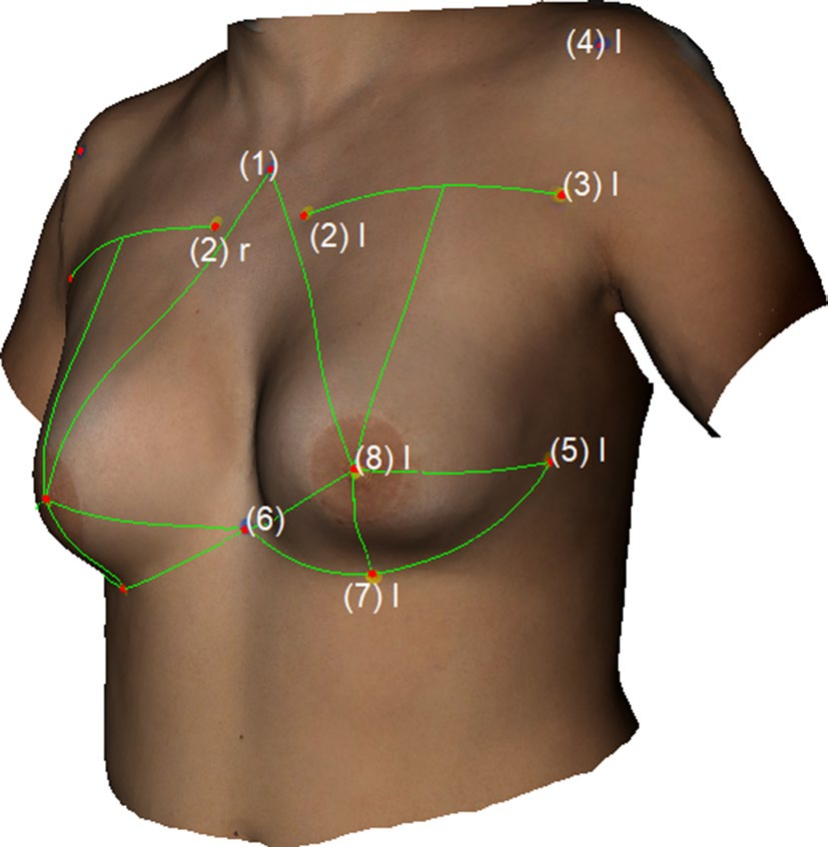
Automatic measurements. Appearance of a 32-year-old patient 12 years after bilateral permanent silicone gel breast implant installation; 225 cc (R)/200 cc (L); mastopexy R + L; lateral view

**Fig. 4:**
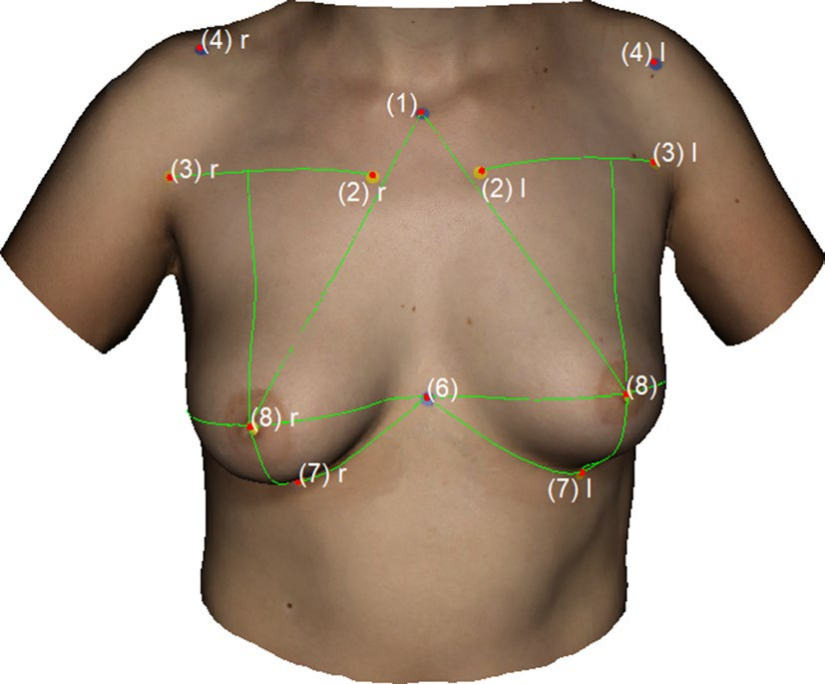
Automatic measurements. Appearance of a 22-year-old patient 2 years after lipofilling (L) in three stages; total transplanted volume, 540 cc (L); frontal view

**Fig. 5:**
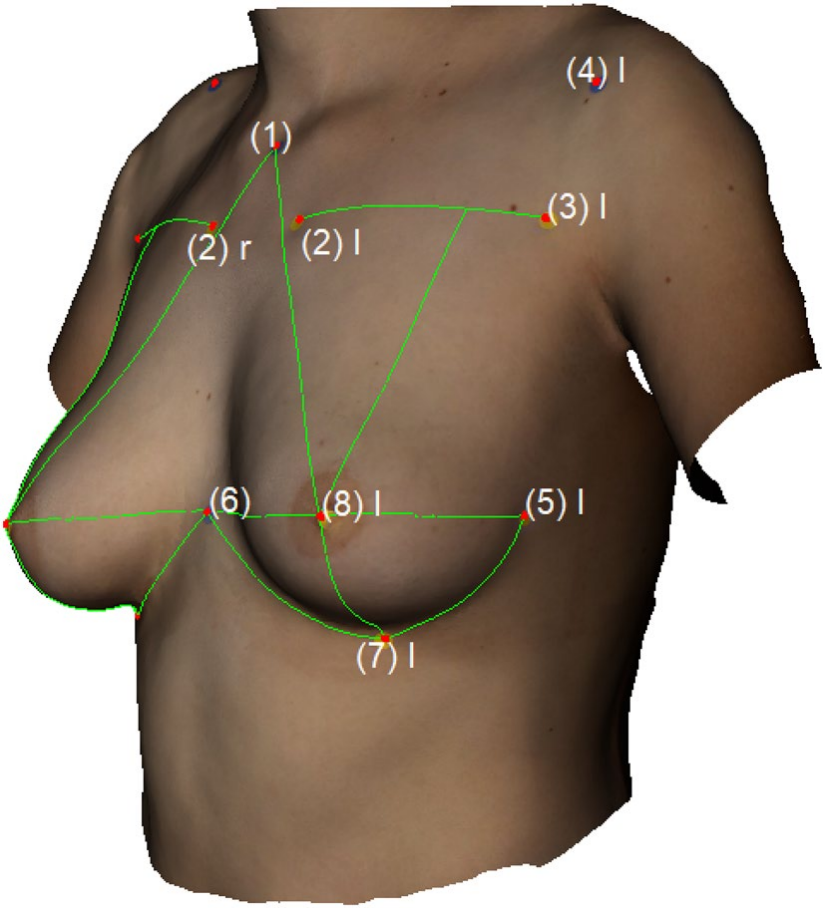
Appearance of a 22-year-old patient 2 years after lipofilling (L) in three stages; total transplanted volume, 540 cc (L); lateral view

The following measurements were obtained: (1) Sternal Notch to Nipple (SN–N), (2) Inframammary Fold to Nipple (LBP–N), (3) Upper Breast Pole to Nipple (UBP–N), (4) Xiphoid to Nipple (Xi–N), (5) Lateral Breast Pole to Nipple (LaBP–N), (6) Breast Width and (7) Inframammary Fold Length (IMF-Length). See Figs. 2, 3, 4, 5 for more details. To perform measurement (3), a guideline representing the UBP was created through landmarks (2) and (3). The shortest UBP–N distance represents measurement (3). The described measurements were used to calculate the Symmetry Index (SI) according to our previously described protocol [13]. It combines measurements (1)–(7) into one analytical value. The anthropometric measurements were obtained manually and automatically. For automatic measurements, the previously developed software was used. For manual measurements, a regular tape measure was used.

### Manual anthropometric measurements

The use of 3D surface imaging for assessment of the breast region has gained importance in the 2 past decades [18–24]. However, analogous manual measurement is still a well-validated, standardized method for outcome assessment in breast surgery [1]. To validate our novel software, we compared manual and automated measurements obtained from the same patient. For each breast, seven distinct anthropometric measurements were obtained. Each distance was measured from the sticker’s midpoints. Manual measurements were obtained by a healthcare professional, and a board-certified plastic surgeon checked and revised them. A regular tape measure was used to obtained analogous anthropometric measurements. For measurement (3), the guideline representing the UBP was created through landmarks (2) and (3) using a linear ruler.

After obtained the measurements, the manual SI was calculated for every patient.

### Automatic anthropometric measurements

**Fig. 6:**
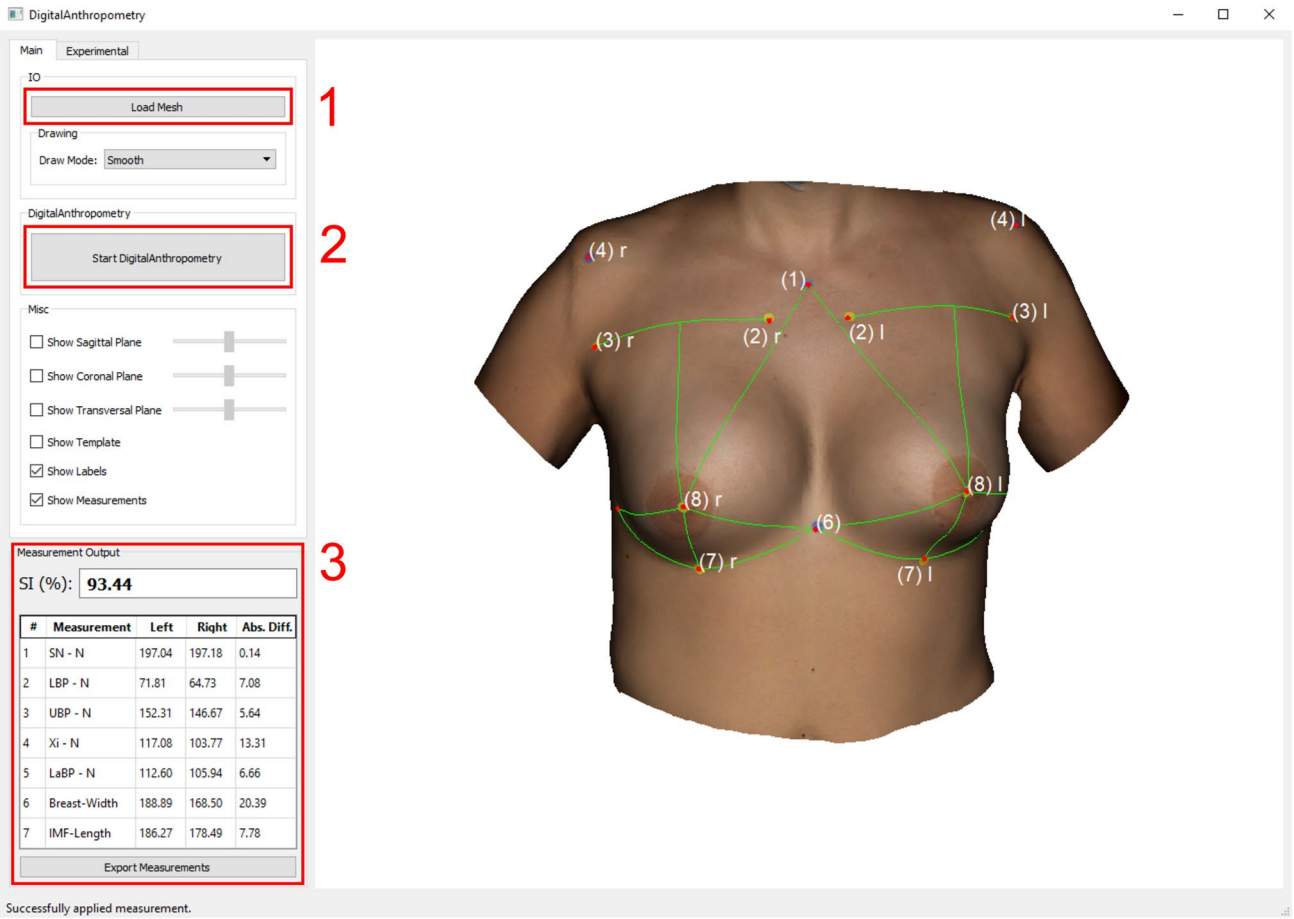
The graphical user interface of the proposed software and main steps to obtain the desired measurements shown in red: (1) Load a surface mesh, (2) start the fully automatic algorithm for digital anthropometric measurements by clicking the second button, and finally (3) analyze or export the obtained measurements as well as the SI. The software is intended for research purposes and is not commercially available.

In addition to the aforementioned manual measurements, fully automatic measurements were obtained from all patients using our software, which has been previously described elsewhere [13]. The software enables the examiner to use all common types of 3D file formats for digital anthropometry. In the present trial, Wavefront OBJ files and JPEG files were used for texture. The pre- and postprocessing of the 3D model were performed as previously described [13]. The prototype graphical user interface of the software is shown in Fig. 6. As soon as a 3D dataset is loaded, the software algorithm automatically determines all landmarks by detecting the colored stickers and obtains the measurements as described.

### Statistical analysis

IBM SPSS 25 was used for statistical analysis. Per patient, 14 manual and 14 computer-based measurements were obtained. The mean values of the measurements for the right and left breast (*n* = 322) were calculated. Then, they were analyzed using the Wilcoxon signed-rank test to compare the accordance of the manually measured values with the automatically measured values for (1)–(7) and the SI (Table 1). A total of 322 measurements and the SI were compared.

**Table 1:** Breast R + L

Test statistics								
	(1) SN–N manual/automatic	(2) LBP–N manual/automatic	(3) UBP–N manual /automatic	(4) Xi–N manual/automatic	(5) LaBP–N manual/automatic	(6) IMF-Length manual/automatic	(7) Breast width manual/automatic	(8) Si automatic/manual
Asymp. Sig. (2-tailed)	0.10	0.17	0.04	0.83	0.75	0.91	0.60	0.10

Manual and automatic measurements and Symmetry Index compared by Wilcoxon Signed Ranks Test; *IBM SPSS 25* was used for data analysis

## Results

The cohort included eleven patients who underwent successful lipofilling and twelve patients who underwent successful installation of a silicone gel implant.

Three of the 11 women who underwent lipofilling suffered from Poland syndrome. Three others suffered from tuberous breast deformity, and the remaining five suffered from Amazon syndrome. In this lipofilling group, three and eight women underwent unilateral and bilateral lipofilling, respectively. Seven patients underwent a single-stage therapy, two patients underwent a two-stage therapy, and two patients underwent a three-stage therapy. Among these women, one patient underwent unilateral mastopexy, and one patient underwent bilateral mastopexy. Four patients underwent additional unilateral mammary reduction surgery. According to the Regnault classification [25], 5 of the 11 women who had undergone lipofilling had nonptotic breasts bilaterally. Three patients had bilateral grade I ptosis. One patient had bilateral grade II ptosis. One patient had unilateral grade II ptosis on the left side. One patient had unilateral grade I ptosis on the right side. The mean added breast volume was *M* = 587 cc (SD = ± 308 cc). The added breast volume was measured based on the volume injected intraoperatively.

The 12 women who underwent successful implant installation included 1 patient with Poland syndrome, 6 patients with tuberous breast deformity, and 4 patients with Amazon syndrome. The silicone gel implant was placed unilaterally in four patients and bilaterally in seven patients. Among these women, two patients underwent additional unilateral mastopexy, three patients underwent additional bilateral mastopexy, and seven did not undergo supplementary mastopexy. One patient underwent additional unilateral mammary reduction surgery. According to the Regnault classification [25], the implant group included nine patients with bilateral nonptotic breasts. Two patients had bilateral grade IV ptosis. One patient had grade I ptosis on the right and grade III ptosis on the left. The implant was placed epipectorally in five patients and subpectorally in six patients. The mean added breast volume was *M* = 388 cc (SD = ± 132 cc).

The mean age was *M* = 30 years (SD = ± 6), the mean height was *M* = 166 cm (SD = ± 7 cm), the mean weight was *M* = 66 kg (SD = ± 13 kg), and the mean BMI was *M* = 24 (SD = ± 4).

Table 2 shows the descriptive statistics for measurements (1)–(7).

**Table 2:** Descriptive statistics

	*N*	Minimum	Maximum	Median	Std. deviation
SN–N (R + L) automatic	23.0	19.0	27.1	21.5	2.3
SN–N (R + L) manual	23.0	19.0	28.3	21.8	2.3
LBP–N (R + L) automatic	23.0	6.3	13.2	8.5	1.6
LBP–N (R + L) manual	23.0	6.5	12.5	8.5	1.5
UBP–N (R + L) automatic	23.0	12.7	21.2	16.1	2.1
UBP–N (R + L) manual	23.0	12.0	23.0	16.8	3.1
Xi–N (R + L) automatic	23.0	9.5	15.8	12.4	1.4
Xi–N (R + L) manual	23.0	9.3	16.0	12.3	1.5
LaBP–N (R + L) automatic	23.0	9.6	16.0	11.8	1.8
LaBP–N (R + L) manual	23.0	9.5	16.0	12.0	1.8
IMF-Length (R + L) automatic	23.0	16.1	27.7	20.8	2.7
IMF-Length (R + L) manual	23.0	16.5	28.0	21.0	2.8
Breast width (R + L) automatic	23.0	19.1	30.2	24.9	2.7
Breast width (R + L) manual	23.0	19.3	30.8	24.8	2.9
SI manual	23.0	79.0	96.0	93.0	3.5
SI automatic	23.0	79.0	96.0	93.0	3.5
Valid *N* (listwise)	23.0				

Values for manual and automatic measurement (1–7) and SI, automatic and manual; mean values for left and right breast were used; IBM SPSS 25 was used to create the table

The values for (1) SN–N (R + L) for the left and right breasts did not differ significantly between the manual (*M* = 21.8) and automatic (*M* = 21.5) methods (Wilcoxon signed-rank test;* p* = 0.10,* n* = 23) (Table 1).

The values for (2) LBP–N (R + L) did not differ significantly between the manual (*M* = 8.5) and automatic (*M* = 8.5) methods (Wilcoxon signed-rank test; *p* = 0.17, *n* = 23) (Table 1).

The values for (3) UBP–N (R + L) did differ significantly between the manual (*M* = 16.8) and automatic (*M* = 16.1) methods (Wilcoxon signed-rank test; *p* = 0.04, *n* = 23) (Table 1).

The values for (4) Xi–N (R + L) did not differ significantly between the manual (*M *= 12.3) and automatic (*M* = 12.4) methods (Wilcoxon signed-rank test; *p* = 0.83, *n* = 23) (Table 1).

The values for (5) LaBP–N (R + L) did not differ significantly between the manual (*M* = 12.0) and automatic (*M* = 11.8) methods (Wilcoxon signed-rank test; *p* = 0.75, *n* = 23) (Table 1).

The values for (6) IMF-Length (R + L) did not differ significantly between the manual (*M* = 21.0) and automatic (*M* = 20.8) methods (Wilcoxon signed-rank test; *p* = 0.91, *n* = 23) (Table 1).

The values for (7) breast width (R + L) did not differ significantly between the manual (*M* = 24.8) and automatic (*M* = 24.9) methods (Wilcoxon signed-rank test; *p* = 0.60, *n* = 23) (Table 1).

The values for the SI did not differ significantly between the manual (*M* = 93.0) and automatic (*M* = 93.0) methods (Wilcoxon signed-rank test; *p* = 0.10, *n* = 23) (Table 1).

The Wilcoxon signed-rank test revealed no significant difference between the automatic and manual methods in measurements (1), (2), (4), (5), (6) or (7). A statistically significant difference between the automatic and manual methods was found in measurement (3). The SI showed no statistically significant difference between the automatic and manual methods.

## Discussion

The proposed method has proven to be precise, as all measurements except measurement (3) showed robust agreement. Nevertheless, the proposed method has limitations, necessitating further discussion.

Of note is the statistically significant variance found for measurement (3) (UBP–N). The reason for the detected difference is the precision of the automated measurements. The software measures UBP–N as the shortest distance from an intersection point along a guideline between the MUBP and the LUBP and a plane through the nipple that is shifted in parallel to the sagittal plane. In the clinical examination, however, the examiner had to detect this point on the guideline using their own estimation. For manual measurements, the guideline between the MUBP and LUBP was applied using a ruler to obtain measurement (3). As the software’s algorithm defines the point more accurately and detects the shortest distance precisely, the values differed significantly. As measurement (3) did not demonstrate statistical importance, the value was excluded from future investigations.

Despite the disagreement found in measurement (3), all remaining measurements, including the SI, showed agreement between the manual and automatic methods.

Other limitations involve the novel method of defining the sagittal plane. In our previous investigations, 360° models were used to define the sagittal plane. The previous method used an extra landmark. In the present trial, 180° models were used. This was possible due to improvements to our algorithm. The novel method defines the sagittal plane through landmarks (1) and (6) and a point between (1) and (6), which is required for definition of the upper breast pole. This method showed robust results in previous investigations [11]. Therefore, 180° models could be used for measurements. Consequently, the additional landmark used previously was excluded. This may limit the accuracy of the measurements. However, similar methods have been introduced using 180° models, showing accurate results [11]. Further studies could compare the results of anthropometric measurements between 360° and 180° models.

Despite technical advances, landmark detection is still a challenge. The described landmarks are detected using colored stickers. On one hand, this technique guarantees reliable results in landmark detection, as a majority of the landmarks are detected by palpating solid structures. On the other hand, this is a very time-consuming process and requires a trained examiner. The described technique is still used due to the lack of a generally accepted method of automated landmark detection without the use of stickers. However, the systemic errors in landmark detection are being reduced through the current software by automating sticker detection. Future analyses should investigate the interrater reliability of the technique described. Additionally, further sophisticated methods will be required to ease the process of automated digital anthropometry.

To date, the use of 3D surface imaging has been undisputed. However, despite the numerous advantages of 3D surface imaging in breast assessment, there are some limitations to its use. With the present trial, we aim to raise awareness of these limitations as well as the uncritical use of the regular software provided. By validating our method, we attempt to contribute to advancements in digital breast assessment.

## Conclusion

By validating the software we previously introduced, we aim to enable advancements in digital anthropometry. The proposed data allow us to improve the usage of our method. The novel software enables the use of 180° models for measurements. Six measurements and the SI showed robust agreement between the manual and automatic methods and can be utilized for symmetry assessment in future investigations.
