# Correction to: New aspects in digital breast assessment: further refinement of a method for automated digital anthropometry

**DOI:** 10.1007/s00404-021-06275-5

**Published:** 2021-10-15

**Authors:** Robin Hartmann, Maximilian Weiherer, Daniel Schiltz, Magnus Baringer, Vivien Noisser, Vanessa Hösl, Andreas Eigenberger, Stephan Seitz, Christoph Palm, Lukas Prantl, Vanessa Brébant

**Affiliations:** 1 University Center of Plastic, Aesthetic, Hand and Reconstructive Surgery, University Hospital Regensburg, Franz-Josef-Strauß-Allee 11, 93053 Regensburg, Germany; 2 Regensburg Medical Image Computing (ReMIC), Ostbayerische Technische Hochschule Regensburg (OTH Regensburg), Regensburg, Germany; 3 Department of Obstetrics and Gynecology, Caritas Hospital St. Josef, University of Regensburg, Regensburg, Germany; 4 Regensburg Center of Biomedical Engineering (RCBE), OTH Regensburg and Regensburg University, Regensburg, Germany; 5 Faculty of Mechanical Engineering, Ostbayerische Technische Hochschule Regensburg (OTH Regensburg), Regensburg, Germany

## Correction to: Archives of Gynecology and Obstetrics (2021) 303:721–728 10.1007/s00404-020-05862-2

The article “New aspects in digital breast assessment: further refinement of a method for automated digital anthropometry” written by Robin Hartmann, Maximilian Weiherer, Daniel Schiltz, Magnus Baringer, Vivien Noisser, Vanessa Hösl, Andreas Eigenberger, Stephan Seitz, Christoph Palm, Lukas Prantl and Vanessa Brébant was originally published electronically on the publisher’s internet portal on November 12, 2020 without open access. With the author(s)’ decision to opt for Open Choice the copyright of the article changed to © The Author(s) 2020 and the article is forthwith distributed under a Creative Commons Attribution 4.0 International License, which permits use, sharing, adaptation, distribution and reproduction in any medium or format, as long as you give appropriate credit to the original author(s) and the source, provide a link to the Creative Commons licence, and indicate if changes were made. The images or other third-party material in this article are included in the article’s Creative Commons licence, unless indicated otherwise in a credit line to the material. If material is not included in the article’s Creative Commons licence and your intended use is not permitted by statutory regulation or exceeds the permitted use, you will need to obtain permission directly from the copyright holder. To view a copy of this licence, visit http://creativecommons.org/licenses/by/4.0/. Open Access funding enabled and organized by Projekt DEAL.

The original article has been updated.

